# Characterization of culturable bacterial endophytes and their capacity to promote plant growth from plants grown using organic or conventional practices

**DOI:** 10.3389/fpls.2015.00490

**Published:** 2015-07-10

**Authors:** Ye Xia, Seth DeBolt, Jamin Dreyer, Delia Scott, Mark A. Williams

**Affiliations:** ^1^Department of Horticulture, University of KentuckyLexington, KY, USA; ^2^Department of Entomology, University of KentuckyLexington, KY, USA

**Keywords:** organic farming, endophyte, diversity, phytobiome, agroecosystems

## Abstract

Plants have a diverse internal microbial biota that has been shown to have an important influence on a range of plant health attributes. Although these endophytes have been found to be widely occurring, few studies have correlated agricultural production practices with endophyte community structure and function. One agricultural system that focuses on preserving and enhancing soil microbial abundance and biodiversity is organic farming, and numerous studies have shown that organically managed system have increased microbial community characteristics. Herein, the diversity and specificity of culturable bacterial endophytes were evaluated in four vegetable crops: corn, tomato, melon, and pepper grown under organic or conventional practices. Endophytic bacteria were isolated from surface-sterilized shoot, root, and seed tissues and sequence identified. A total of 336 bacterial isolates were identified, and grouped into 32 species and five phyla. Among these, 239 isolates were from organically grown plants and 97 from those grown conventionally. Although a diverse range of bacteria were documented, 186 were from the Phylum Firmicutes, representing 55% of all isolates. Using the Shannon diversity index, we observed a gradation of diversity in tissues, with shoots and roots having a similar value, and seeds having the least diversity. Importantly, endophytic microbial species abundance and diversity was significantly higher in the organically grown plants compared to those grown using conventional practices, potentially indicating that organic management practices may increase endophyte presence and diversity. The impact that these endophytes could have on plant growth and yield was evaluated by reintroducing them into tomato plants in a greenhouse environment. Of the bacterial isolates tested, 61% were found to promote tomato plant growth and 50–64% were shown to enhance biomass accumulation, illustrating their potential agroecosystem application.

## Introduction

Plant endophytes have been defined as organisms that colonize internal plant tissues without causing apparent harm to the host (Petrini, 1991). The association between plants and bacterial endophytes developed very early in evolution (Saikkonen et al., 2004; Zhao and Qi, 2008; Kawaguchi and Minamisawa, 2010), and it is likely that this association occurs in all plant species (Rosenblueth and Martínez-Romero, 2006). In these mutualistic interactions, the plant host provides diverse protective niches for endophytic organisms, and endophytes can produce useful metabolites and signals (Gary, 2003; Rosenblueth and Martínez-Romero, 2006) which can increase nutrient uptake (Ramos et al., 2011), modify plant growth, development and biomass (Compant et al., 2005; Hardoim et al., 2008), induce resistance to pathogens (Sturz and Matheson, 1996; Nagarajkumar et al., 2004; Padgham et al., 2005) and insects (Azevedo et al., 2000), and increase resistance to osmotic stress (Sziderics et al., 2007), heavy metals (Rajkumar et al., 2009), contaminated chemicals (Siciliano et al., 2001), and other abiotic factors. Plant–endophyte interactions have also been shown to have critical impacts on the integrity, proper function and sustainability of agro-ecosystems (Barac et al., 2004; Nagarajkumar et al., 2004).

Although originally documented long ago (Perotti, 1926), there are many aspects of plant–endophyte associations that remain poorly understood. For example, the physiological, genetic and molecular mechanisms utilized during this inter-organism association, both by the host plant to select mutualistic rather than pathogenic associates, and also by the cognate microbe during association is unclear. Additionally, although there are examples of endophytes colonizing aerial plant organs, the majority of studies have focused on root endophytic associations (Lundberg et al., 2012, 2013; Romero et al., 2014). Importantly, the environmental factors and farming practices that affect endophyte community diversity, and the mechanisms in which plant–endophyte associations occur in agroecosystems are not well studied (Rosenblueth and Martínez-Romero, 2006; Reinhold-Hurek and Hurek, 2011).

Organic farming has been one of the fastest growing segments of agriculture in the United States since the early 1990s, and total cropland in certified organic production from 2000 to 2011 increased by 153% to over 3 million acres (U.S. Department of Agriculture [USDA], 2013). Among the defining characteristics of organic systems are the integration of practices that aim to increase soil quality and optimize nutrient cycling, while excluding synthetically derived pesticides, and petroleum based fertilizers. From the beginning of this farming movement, one of the central philosophies has been to utilize techniques, such as adding compost, manure and cover crop amendments to build soil humus in order to optimize soil microbial health and biodiversity (Zarb et al., 2005; Heckman, 2006; Thilmany, 2006). Multiple studies have compared conventional and organic farming systems. These studies reveal that organic practices influence soil, and notably are linked to higher soil microbial populations, activity and community diversity (Monokrousos et al., 2006; Esperschütz et al., 2007; Fließbach et al., 2007; Araújo et al., 2009; Li et al., 2012). To conserve system biodiversity, including soil microbes, organic practices exclude conventional pesticides (e.g., fungicides, bactericides, insecticides, herbicides) and effectively reduce their impact on non-target organisms (Kuepper and Gegner, 2004). An outlying question is whether organic practices could positively influence the plant bacterial endophyte community (phytobiome). Here, we take initial steps to address this question by examining the culturable endophyte phytobiome from four economically important vegetable crops grown under organic or conventional management practices. The goal of this study was to advance our understanding of whether plant endophyte communities are impacted by production system.

## Materials and Methods

### Plant and Soil Sample Collection

Whole plants of sweet corn (*Zea mays* L.), tomato (*Solanum lycopersicum* L.), watermelon (*Citrullus lanatus* Thunb.), and bell pepper (*Capsicum annuum* L.) were collected during the summer of 2012 at the University of Kentucky Horticulture Research Farm in Lexington, KY (lat. 38° 3′N, long. 84° 30′W). The soil on the farm was a Maury silt loam series (0–2% slope), which is a fine, mixed mesic Typic Alfisol. For each species, five fruiting stage plants were collected separately on a section of the research farm that has been managed using typical conventional farming practices appropriate for the region, as outlined in the University of Kentucky Vegetable Production Guide for Commercial Growers (University of Kentucky, 2014) for over 30 years, or on a section of the farm that has been managed using USDA certified organic management practices according to the National Organic Program’s Organic Standards, since 2005 (U. S. Department of Agriculture [USDA], 2014). Representative soil samples in the area around the root zone from each plant were collected at a depth of 0–15 cm and within 30 cm of the plant stem/root interface. The soil samples were analyzed for pH, and other soil chemical parameters by the University of Kentucky Regulatory Service Soil Testing Lab according to (Soils and Plant Analysis Council, 2000).

### Isolation of Bacterial Endophytes from Shoot, Root, and Seed Tissue

Shoot (collectively the leaf and stem) and root segments were prepared as described in Xia et al. (2013). Briefly, segments of ∼1–1.5 cm in length with similar weight were hand cut from plant tissue samples and washed with deionized water (dH_2_O) prior to sequential rinsing with 95% ethanol (EtOH) for 2 min. Finally, segments were immersed in a solution of 30% Clorox (household grade) bleach in sterile dH_2_O for 20 min, followed by a series of five rinses with sterile dH_2_O. Seeds were excised from their respective fruits, cut in half, and surface-sterilized as above. All surface-sterilized plant specimens were placed separately on culture plates containing YPDA medium (Clontech Laboratories, Inc., Mountain View, CA, USA). Nystatin (Fisher Scientific, Bridgewater Township, NJ, USA) was added to the YPDA medium to a final concentration of 100 μg/ml to prevent fungal growth. Aliquots of the final dH_2_O rinse were plated on YPDA plates to verify that no surface bacteria were present. Equal numbers of tissue samples from plants grown in the two systems were incubated at 26°C for 3–5 days on YPDA plates and the endophytic bacteria emerging from the cut ends of the samples onto the culture plates were selected and streaked on YPDA plates separately. Single colonies were selected from each formation and sub-cultured separately (Long et al., 2008). This procedure was repeated 2–3 times in order to obtain a pure isolate.

### DNA Extractions, 16S rDNA Gene Amplification, Sequencing, and Species Identification

Individual colonies were isolated from plates using a sterile pipette tip and transferred to a streak plate to confer uniformity, and grown separately in liquid culture (YPD broth; Clontech Laboratories Inc., Mountain View, CA, USA) overnight at 26°C on a rotary shaker at 200 rpm. From the resulting pelletized cells, DNA was extracted using a Zymo Research fungal/bacterial DNA miniprep kit (Zymo Research, Irvine, CA, USA). Amplification of the 16S rDNA (50 μl reaction) included 3 μl DNA template (1–20ng), 100 μM of primers 27f (5′-GAGTTTGATCCTGGCTCA-3′) and 1498r (5′-ACGGCTACCTTGTTACGACTT-3′), which are complementary to the conserved regions at the 5′-and 3′- ends of the *Escherichia coli* 16s rRNA gene at the positions of 9–27 and 1477–1498 respectively (Lane, 1991; Reddy et al., 2000), 3 mM Mgcl2, 3 mM dNTPs, 5 μl of Taq buffer, and 1 U Taq DNA polymerase (Fermentas, Inc., Hanover, MD, USA). PCR amplification was performed on an icycler PCR machine (Bio-Rad Laboratories, Berkeley, CA, USA), with the initial denaturation at 94°C for 5 min, followed by 50 cycles of amplification (94°C for 1 min, 54°C for 1 min, 72°C for 2 min) and an extension step (72°C for 5 min). The PCR products were purified using a Fermentas GeneJET PCR purification kit (Fermentas, Inc., Hanover, MD, USA), quantified on a nanodrop spectrophotometer and sequenced by Elim Biopharm Inc. (Hayward, CA, USA). Sequences were edited manually or by Bioedit Sequence Alignment Editor, and subjected to BLASTn searches in the NCBI and BIBI Databases (Devulder et al., 2003; Mignard and Flandrois, 2006). The top database hits were used to identify the most probable taxonomic resolution to species level with at least a 95% confidence interval.

Bacterial species derived from specific host plants were grouped into their higher taxonomic level (Phylum level) so that the Phylum frequencies could be evaluated based on their tissue and farming system (conventional vs. organic management) distribution. Species diversity and the relative species abundance of the 32 microbial species identified in this study were calculated using the Shannon diversity index (Bowman et al., 1971). Prior to diversity analysis, samples that did not test positive for any of the 32 bacterial species were removed and the data were square root transformed. The effects of farming method, plant species, and tissue type on the abundance and diversity of endophytic bacteria in the four plant species was compared using two-way PERMANOVAs with 9999 permutations in the “vegan” package (Oksanen et al., 2013) of R 3.0.1 (R Core Development Team, 2013). Bacterial species presence was summed across tissue types within the five replicate plants of each species to test the overall effect of farming method and their interaction with plant species, while non-aggregated bacteria counts were used to analyze the effects of farming method by tissue type within a plant species, and tissue types by farming method across plant species.

*P*-values for the bacterial species abundance based on plant type and production system (conventional or organic) analysis were calculated using the Mann–Whitney pairwise *post hoc* test through the PAST software program (http://folk.uio.no/ohammer/past/, version 3.05, University of Oslo, Oslo, Norway). The experimental design was a factorial arrangement comparing five replications of the four plant species, each grown under two production systems: organic or conventional.

### Screening for Plant Growth Promoting Bacterial Isolates

Tomato seeds (cultivar Rutgers, Ferry Morse, MA, USA) were washed with 95% EtOH in dH_2_O for 2 min and then treated for 25 min in a solution of 30% Clorox, 5% sodium dodecyl sulfate (SDS) solution in dH_2_O. They were then rinsed five times with sterilized dH_2_O and incubated at 4°C for 24 h. Seed sterility was assessed by plating aliquots of the seeds on YPDA plates and confirming no microbial growth. Individual colonies of endophytes isolated from plants grown in both production systems were grown in YPD broth medium overnight at 26°C on a rotary shaker at 200 rpm to the log phase of the bacteria growth (OD600 = 0.6). The sterilized tomato seeds were added to the bacteria broth, with a final concentration of approximately 10e8 bacteria per seed, and grown at 26°C on a rotary shaker at 200 rpm for an additional 24 h. The bacteria-treated tomato seeds were then sown into 15 cm diameter pots containing autoclaved Pro-Mix potting media (Premier Horticulture, Inc., Quakertown, PA, USA), and sterilized seeds without the bacterial treatment were sown as controls in separate pots. The plants were grown in a green house for 60 days with constant temperatures of 28°C, and 16 h of light followed by 8 h of dark. The above soil height, fresh weigh and dry weight of the tomato plants were recorded and the comparison of height, fresh weight and dry weight between plants treated with each individual bacterial species were compared with the untreated control plants by the Student *t*-test at a 95% confidence level. All the experiments contained three replicates (*n* = 3).

## Results

### Endophyte Community Diversity and Abundance

**FIGURE 1:**
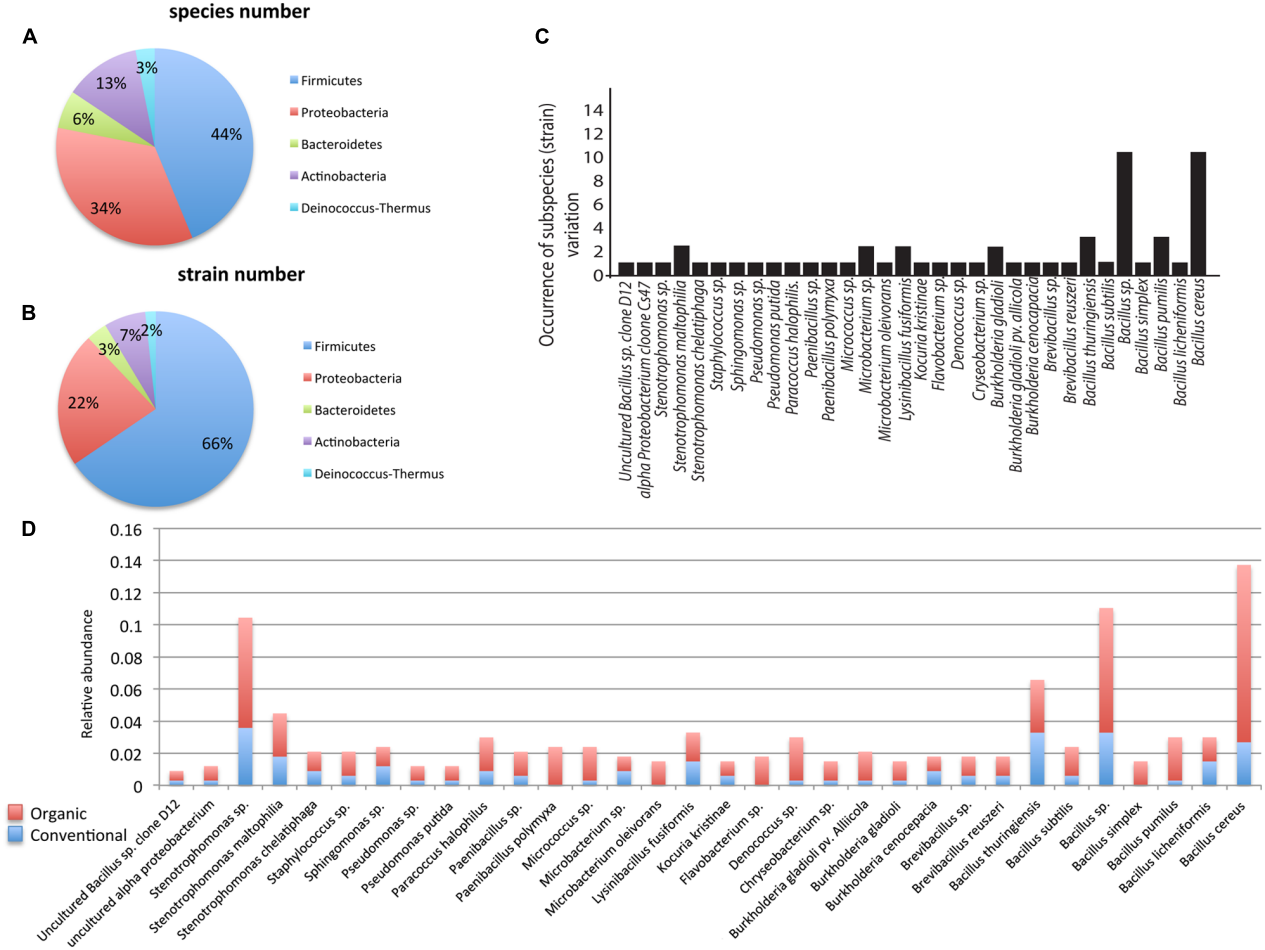
**Taxonomic distribution of endophytic bacteria isolated from corn, watermelon, pepper, and tomato grown under two production systems: organic and conventional.** The distribution of species (*n* = 32; **A**), strains (*n* = 58; **B**). All data reflect the total from all four plants and both production systems. Distribution of strains beyond the species level was examined **(C)** where bars represent the occurrence of genotypes/subspecies (*n* = 58) among the 336 different bacterial isolates from the four plants in this study. Relative species abundance was examined between the two production systems **(D)**. Data are broken into organic or conventional production systems in each species.

A total of 336 endophytic bacterial isolates were cultured from tomato, corn, watermelon, and pepper plants that were grown in either a conventional or a certified organic production system. Through 16S rDNA sequence identification we demonstrated that these plant-associated endophytic bacteria could be classified into 32 distinct species (summarized in **Figure 1**). Further classification showed that the 32 species belonged to five bacterial phyla. The most commonly isolated bacteria were in the phylum Firmicutes, which included 14 species and 186 isolates and constituted ∼44% of the total species types in this study (**Figures 1A,B**). Species in phylum Firmicutes included *Bacillus cereus, B. licheniformis, B. pumilus, B. simplex, Bacillus* sp., *B. subtilis, B. thuringiensis, Brevibacillus reuszeri, Brevibacillus* sp., *Lysinibacillus fusiformis, Paenibacillus polymyxa, P.* sp., *Staphylococcus* sp., and uncultured *Bacillus* sp. Other phyla identified included Proteobacteria, Actinobacteria, Bacteroidetes, and Deinococcus-Thermus. Proteobacteria was the second most abundant phylum and contained 105 isolates and 11 species including *Burkholderia cenocepacia, B. gladioli, B. gladioli* pv. *allicola, Paracoccus halophilus, Pseudomonas putida, P.* sp., *Sphingomonas* sp., *Stenotrophomonas chelatiphaga, S. maltophilia, Stenotrophomonas* sp., and uncultured *alpha proteobacterium*. The phylum Actinobacteria included four species: *Kocuria kristinae, Microbacterium* sp., *M. oleivorans, Micrococcus* sp. The phylum Bacteroidetes included *Chryseobacterium* sp. and *Flavobacterium* sp. The phylum Deinococcus-Thermus had the lowest abundance and included only one species, *Deinococcus* sp.

Identification of bacterial isolates below the species level, referred herein as genotypes, revealed that the species *Bacillus* sp. and *B. cereus* contained the highest number of genotypes, with 10 each, followed by *B. pumilus* and *B. thuringiensis*, which contained three genotypes each, while the other 28 species only contained 1–2 genotypes each (**Figure 1C**). Interestingly, examination of the distribution pattern within the endophytic bacterial isolates showed that 10 species comprised ∼61% of the total 336 isolates, with the most abundant being *B. cereus*, followed by *Bacillus* sp., *Stenotrophomonas* sp., *B. thuringiensis, Stenotrophomonas maltophilia, Lysinibacillus fusiformis, Deinococcus* sp., *B. licheniformis, B. pumilus, Paracoccus halophilus* (**Figure 1C**).

### Endophyte Relative Species Abundance and Diversity Associated with Farm Management Practices

Of the 336 total isolates, 71% (239 isolates) were obtained from plants that were grown using organic farming practices, and 29% (97 isolates) from plants grown using conventional farming practices. The relative species abundance of bacterial endophytes was significantly higher in plants through organic practices than conventional practices (**Figure 1D**, *P* < 0.05, **Table 1**). A total of five phyla and 28 species were common to both systems (**Figure 1D**). Unique to the organically grown plants were the species *B. simplex*, *Flavobacterium* sp., *Microbacterium oleivorans, Paenibacillus polymyxa* (**Figure 1D**). Collectively there were 32 species isolated from plants grown organically, and 28 species from plants grown conventionally (**Figure 1D**). The Shannon diversity index value revealed that the species diversity of bacterial isolates were higher in all of the organically managed plants, although this difference was only significantly higher in pepper (**Table 2**). Importantly, the endophyte diversity was significantly higher among all crops grown organically versus those grown using conventional practices (**Table 2**, *P* = 0.049). Among the four plant species evaluated in this study, the diversity index was highest in melon, and lowest in the bell pepper, and this trend was consistent across management systems (**Table 2**).

**Table 1:** Bacterial species abundance based on plant type and production system (conventional or organic).

	Tissue type	Relative abundance	Relative abundance	*P*-value
		Conventional	Organic	
Tomato	Shoot	0.06	0.13	0.04
	Root	0.02	0.08	<0.001
	Seed	0.03	0.01	0.66
	Total	0.11	0.22	<0.01
Corn	Shoot	0.03	0.1	<0.01
	Root	0.02	0.08	<0.001
	Seed	0.01	0.02	0.4
	Total	0.06	0.2	<0.001
Melon	Shoot	0.09	0.15	0.1
	Root	0.01	0.1	<0.001
	Seed	0	0	Na
	Total	0.1	0.25	<0.001
Pepper	Shoot	0.02	0.04	0.08
	Root	0	0.01	Na
	Seed	0	0.01	Na
	Total	0.02	0.05	0.03

*Frequency is shown based on the number of times individual bacteria were cultured from shoot, root or seed tissues in each crop species: tomato, corn, melon, or pepper. P-value was calculated using the Mann-Whitney pairwise post hoc test (PAST version 3.05, University of Oslo, Oslo, Norway). A statistical comparison was not possible when no bacteria species were isolated from individual tissues, and this is noted with na = not applicable. The number of species assessed in this experiment for each tissue type is 32 and the assessment was done from five replicates of each plant species.*

**Table 2:** Shannon diversity indices and the results of two-way PERMANOVAs with 9999 permutations analyzing the effect of farming method and plant tissue origin on the diversity of endophytic microbiota in four plant species.

Plant	Factor	Shannon indices	*F*-value	*N*	*P*-value
Total	Con. vs. Org.	2.96 vs. 3.14	**2.24**	**35 (16, 19)**	**0.049**
Corn	Con. vs. Org.	2.58 vs. 3.01	1.48	16 (6, 10)	0.21
Melon	Con. vs. Org.	2.75 vs. 3.08	1.63	15 (6, 9)	0.17
Pepper	Con. vs. Org.	1.24 vs. 2.34	**3.06**	**10 (3, 7)**	**0.026**
Tomato	Con. vs. Org.	2.57 vs. 2.88	1.42	19 (10, 9)	0.22
All	Shoot vs. Root	3.08 vs. 3.18	1.09	50 (34, 16)	0.37
All	Shoot vs. Seed	3.08 vs. 2.22	**2.69**	**44 (34, 10)**	**0.023**
All	Root vs. Seed	3.18 vs. 2.22	**2.59**	**26 (16, 10)**	**0.017**

*Microbial abundance was summed across tissue types within the five replicate plants of each species to test the overall effect of farming method and its interaction with plant species (Plant = “Total”), while non-aggregated microbe counts were used to analyze the effects of farming method by tissue type within a plant species (Plant = species), and tissue types by farming method across plant species (Plant = “All”). Con. is conventional and refers to plants grown using conventional practices, Org. is organic and refers to plants grown using organic practices. Bold identifies significant differences at P < 0.05.*

Soil pH has been found to influence soil bacterial community diversity (Fierer and Jackson, 2006) and bacterial endophyte diversity (Xia et al., 2013). Therefore, we evaluated soil pH for any shifts in correlation with endophyte diversity. Although the soils in both management systems were of the same soil series and on the same farm, differences in soil pH were observed between the different plants and between production systems (**Table 3**). Initially, we analyzed a pairwise comparison of Shannon diversity index values in the four plants in this study versus pH. These data revealed no correlation between soil pH and endophytic bacteria species diversity across all four plant species (data not shown); however, the data showed a consistent pattern within the same plant species, with a higher species diversity for all the organically grown plants versus those grown using conventional practices. This diversity within each plant species seemed to be correlated with pH, in that higher pH values were consistently associated with higher diversity indices (**Table 3**).

**Table 3:** Correlation of soil pH and bacterial diversity as measured by the Shannon diversity index for plants grown under organic or conventional practices.

Crop	Shannon index (Hs)	Soil pH
Org. melon	3.08	5.58
Conv. melon	2.75	5.37
Org. tomato	2.88	5.78
Conv. tomato	2.57	5.37
Org. corn	3.01	6.15
Conv. corn	2.58	5.15
Org. pepper	2.34	5.49
Conv. pepper	1.24	5.27

*Soil samples from each plant were collected from the rhizosphere at a depth of 0–15 cm and within 30 cm of the plant stem/root interface. Shannon index represents bacterial diversity over five replications for each plant type (n = 5). Org., organically grown plants; Conv., conventionally grown plants.*

### Bacterial Community Diversity Associated with Specific Tissues

**FIGURE 2:**
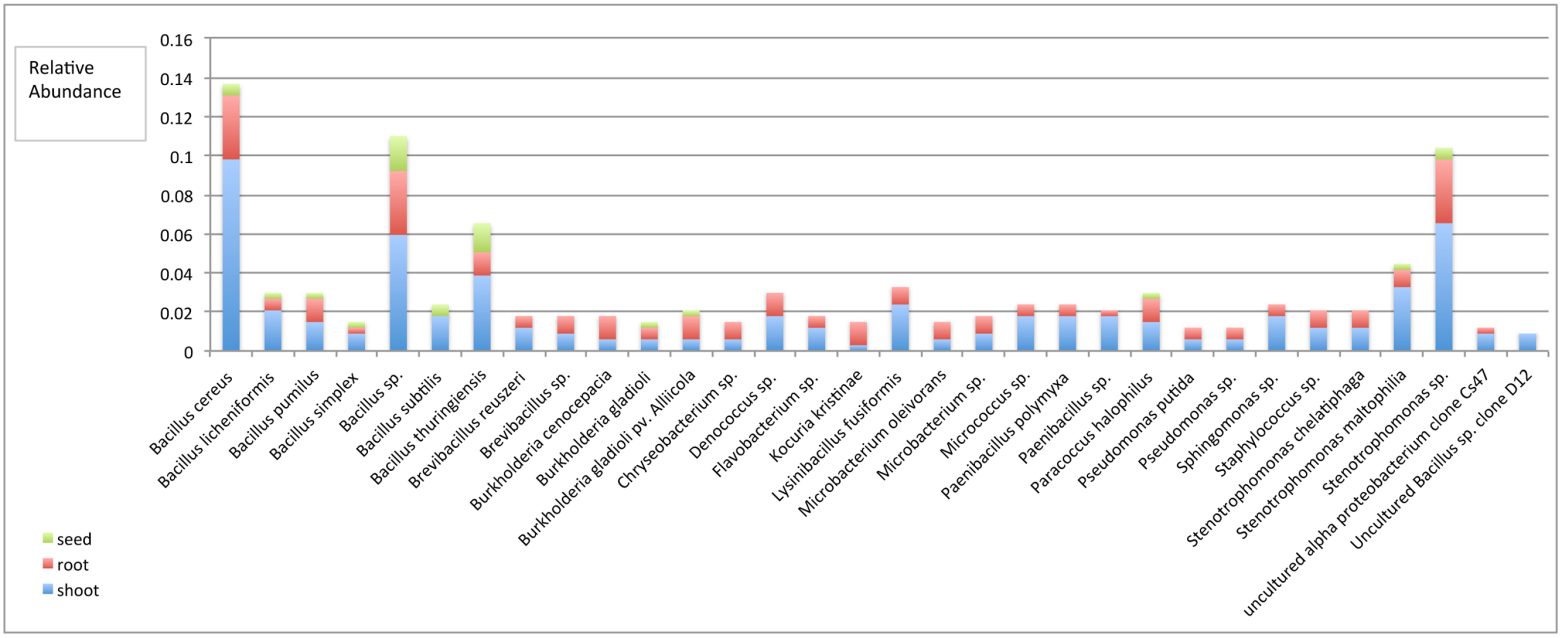
**Relative species abundance among plant tissues.** The frequency at which an isolated endophyte was counted was based on sequence based classification at the species level, which were organized into a meta-analysis bar chart, revealing the relative abundance of specific species among the three tissues. *P* < 0.0001.

Only two phyla, Firmicutes and Proteobacteria, were distributed in all plant tissues, while phylum Actinobacteria, Bacteroidetes, and Deinococcus-Thermus were only found in root and shoot, but not seed tissues. In comparing the Shannon diversity index value of bacterial endophytic communities based on the plant tissue they were extracted from, we found that shoots and roots displayed a similar diversity, but seeds had the lowest diversity (**Table 2**). Notably, of the 32 species only 11 were distributed in all tissues, these including *B. cereus, B. licheniformis, B. pumilus, B. simplex, Bacillus* sp., *B. thuringiensis, Burkholderia gladioli, B. gladioli* pv. *Alliicola, Paracoccus halophilus, Stenotrophomonas maltophilia*, and *Stenotrophomonas* sp. (**Figure 2**). Of the 336 isolates identified within this study, the shoot community had 206 isolates belonging to 32 species; the root community had 106 isolates belonging to 30 species; and the seed community had 24 isolates belonging to 12 species, which meant that there were 61, 32, and 7% bacterial isolates distributed in shoot, root, and seed tissues, respectively (**Figure 2**, see **Table 1** for analysis based on production system).

### Analysis of Endophytic Bacteria and their Capacity to Influence Plant Growth

To advance our understanding of the functionality of the bacteria isolated in this study, we inoculated surface-sterilized tomato seeds with isolated bacteria and evaluated their potential to modulate plant growth and development. As noted above, we isolated a total of 58 different bacterial genotypes that were composed of 32 different species. We therefore screened all 58 genotypes derived from plants grown in both production systems for their capacity to modify plant growth in tomato. Surprisingly, 61% of the bacteria co-inoculated with tomato seeds were found to promote plant growth (aerial height) compared with mock controls; 64% were found to increase the fresh weight of tomato plants, and 50% were found to increase the dry weight of the tomato plants (Supplementary Tables S1–S3, *P* < 0.05, Student *t*-test). Bacteria that increased plant height, fresh weigh and dry weight more than 25% relative to the mock control were mostly in the phylum Firmicutes. Bacterial genotypes that had the highest levels of growth promoting activity included *Bacillus* sp. *clone D12, Bacillus* sp. *DB12, B. cereus isolate T1-9, Brevibacillus* sp. *Z0-YC6800 and Burkholderia gladioli strain PA17.2*, etc. (Supplementary Table S1). These data demonstrate that many of the isolated bacterial species from within plant tissue were capable of positively impacting growth when re-applied to a host plant in isolation.

## Discussion

One of the central management goals of organic farming is to optimize soil microbial community health and diversity. Many of the core practices associated with organic farming, such as organic amendment addition, and humus deposition have been shown to positively impact soil microbial communities, with the ultimate goal of increasing nutrient uptake, disease suppression, and enhancing plant health, potentially through microbial mediated processes. Since it is thought that many endophytes colonize plants from a sub-population of the rhizosphere microbiome (Compant et al., 2010), enrichment of soil microbial communities, as has been observed in organically managed systems, plausibly results in an increased presence of endophyte bacteria, but this remains unclear. Elucidating whether farming practices, such as organic principles versus conventional practices that employ pesticides, can be correlated with modification or enhancement of elements of the plant microbiome is therefore of considerable interest. Numerous evaluations of bacterial endophytes have shown them to be widespread in the plant kingdom (Olivares et al., 1996; Pillay and Nowak, 1997; Siciliano and Germida, 1999; Sessitsch et al., 2004; Hardoim et al., 2008; Lundberg et al., 2012), and consistent with these findings we were able to isolate endophytes from all of the plants in this study (**Table 1**). There were unequal distributions of bacteria isolates among species and genotypes, and the most abundant species were *Bacillus cereus* and *Bacillus* sp., which constituted more than 35% of total genotype variants. To the phylum level, these species all belonged to phylum Firmicutes and Proteobacteria. The Firmicutes constituted ∼44 and 66% of the total bacterial species and genotype variants (**Figures 1A,B**). While we are acutely aware that the distribution and identification of specific bacteria may be a consequence of the cultivability, these findings are consistent with others that have found Firmicutes to be the most predominant phylum of the bacterial endophytes in ginseng (Vendan et al., 2010) and Proteobacteria to be the most abundant phylum of potato (Garbeva et al., 2001).

The relative species abundance of endophytes was consistantly higher in plants grown in the organic than conventional systems (**Figure 1D**; **Table 1**). Of the 32 species isolated, 29 were present in plants grown in both production systems, and three species (*Flavobacterium* sp., *Bacillus simplex, Paenibacillus polymyxa*) were only present in the organically grown plants (**Figure 1D**). *Flavobacterium* has been shown to be enriched in the rhizosphere of a range of plants, including two in this study (bell pepper and tomato), and has been implicated in the induction of plant defense stimulation, and growth promotion (Soltani et al., 2010). It has been proposed that *Flavobacterium* enrichment may be associated with their strong copitrophic properties (Fierer et al., 2012). Although not specifically evaluated in this study, many of the practices associated with organic farming are known to increase soil organic matter (SOM) and this could potentially result in increased *Flavobacterium* abundance. *Bacillus simplex* has recently been shown to have plant growth promoting characteristics, particularly in roots, through the production of siderophores, as well as anti-fungal properties against pathogens such as *Fusarium* (Schwartz et al., 2013). *Paenibacillus polymyxa* has also been shown to have plant growth promoting attributes and increases resistance to certain biotic and abiotic factors (Timmusk et al., 2005). It is beyond the scope of this study to conclusively determine why these three endophytes were only distributed in the organically managed plants, however, prior evidence supports their capacity for positive impact on plant health.

Evaluation of endophyte distribution among plant tissues revealed that only two phyla- Firmicutes and Proteobacteria were distributed in all the tissues, while phylum Actinobacteria, Bacteroidetes, and Deinococcus-Thermus were only found in root and shoot, and not in seed tissues. We caution that culturing techniques exclude a portion of the non-culturable microbiome, which could be revealed through culture-independent approaches using extracted microbial DNA or RNA and analyzing the phylogenetic relevant 16SrRNA or DNA by PCR/RT-PCR and cloning approaches or by bacterial metagenome or metatranscriptome approaches using techniques such as pyrosequencing (Fischer et al., 2012; Lundberg et al., 2013). Out of all the 32 species cultured, only 11 were widely distributed in all the tissues. Shoot tissues contained all 32 species, while roots and seed tissues contained 30 species and 12 species respectively (**Figure 2**). Community diversity analysis revealed distinct difference between the tissues. For example, the bacteria community diversity was slightly higher in roots than shoots, and both tissues had much higher diversity than seed tissues (Hs shoot = 3.08 vs. Hs root = 3.18; Hs shoot = 3.08 vs. Hs seed = 2.22; Hs root = 3.18 vs. Hs seed = 2.22, **Table 2**). These results suggest that endophytic bacteria may display tissue specific distribution, which has been found in other systems (Chi et al., 2005; Sun et al., 2008; Reinhold-Hurek and Hurek, 2011; Thaweenut et al., 2011; Thomas and Reddy, 2013). Analysis of bacterial endophyte diversity revealed a difference based on plant species. For example, bell pepper had the lowest, and watermelon the highest overall diversity (**Table 2**), and these values were consistent across the two production systems. These findings are consistent with those from other studies that indicate that host selection may play a critical role in establishing internal microbiota (Berendsen et al., 2012; Bulgarelli et al., 2012; Neal et al., 2012), and that this selection may be based on host genetic background, as well as developmental and physiological needs (Siciliano et al., 2001; Rosenblueth and Martínez-Romero, 2006).

Analysis of endophyte community diversity among the two production systems revealed a distinct trend: plants grown using organic practices had higher diversity than those grown using conventional practices (Hs organic = 3.14 vs. Hs conventional = 2.96, *P* = 0.049, **Table 2**). This was consistent across all four plants, although the difference varied between the different crops, with pepper having the largest diversity difference, followed by corn, melon and tomato (**Table 2**). One potential explanation for the endophyte diversity difference between the two systems is that there were differences in the rhizosphere microbiome, which could presumably result in differential bacterial colonization into the plant endosphere. Although there were distinct variations in the way the soil was managed between the systems (e.g., tillage technique, cover crop usage, compost and fertility application, and pesticide usage), both soils were very similar (from the same soil series) and of a very high agricultural quality. While it is likely that the soil management practices between these systems could result in multiple soil chemical, physical and biological differences, we chose to simplify our assessment of how soil-level characteristics could be attributed to endophyte differences by focusing on soil pH, which has been reported to be a key determinant of soil bacterial community structure (Fierer and Jackson, 2006). In this study a pairwise comparison between the species diversity and rhizosphere soil pH showed that between the two production systems the organically managed soils consistently had higher pH values, which correlated with higher species diversity in each of the crop plants (**Table 3**). While it is possible that other soil attributes, such as SOM could play a role in rhizosphere and endophyte bacteria diversity, SOM was not shown to be a significant factor in determining endophyte community diversity in switchgrass (Xia et al., 2013). Considering that organically managed soils typically have higher microbial abundance and activity, it is possible that the soil microbial community structure differences between these production systems played a major role in determining endophyte colonization. Pyrosequencing of both the rhizosphere and endophyte bacterial communities, coupled with an extensive soil analysis, is now needed to further elucidate the complexities of how soil properties impact plant microbiome dynamics.

A principal goal of this work was to evaluate the plant growth promoting attributes of the isolated endophytes, which required that we utilize a culturing technique rather than pyrosequencing. Of the 58 endophytic bacterial genotypic variants evaluated in tomato, 61% were found to result in increased growth, and around 50–64% were shown to enhance shoot biomass accumulation compared with the mock control. There could be several possible mechanisms of increasing plant growth and yield, as has been observed in other endophytes, such as increased nitrogen fixation, hormone production, or enhanced phosphate and iron utilization (Compant et al., 2005; Hardoim et al., 2008). Our results showed that the genotypes exhibiting the best growth promotion belonged to phylum Firmicutes, particularly *Bacillus* sp., *Bacillus cereus*, and phylum Proteobacteria, particularly *Burkholderia gladioli*. In addition to growth promotion some of the bacteria isolated in this study have been shown to have other beneficial effects between systems, such as *Bacillus thuringiensis* isolate LDC-391 having specific cytocidal activity against cancer cells (Poornima et al., 2010), and *Bacillus* sp. *DU39* showing extreme virulence to the free-living nematodes (Rae et al., 2010). Collectively, data presented herein supports the notion that production practices can impact plant endophyte communities. Interestingly, our data for the four commercial horticultural crops studied suggested that organic production practices changed or even increased endophyte diversity. Future studies that investigate the same question using pyrosequencing are also needed, as are studies that correlate specific production practices, such as soil disturbance through tillage, organic matter management, and pesticide usage, with endophyte community dynamics. Recent experiments using pyrosequencing have confirmed that organic management practices can indeed result in a modification of soil microbial community structure (Hartmann et al., 2014) but further work correlating these changes with plant endophyte community structure is needed. Additionally, and of critical importance, is the elucidation of the impacts that these endophyte communities could impart of their plant hosts, and how these associations could be preserved or increased to enhance the sustainability of agroecosystems.

## Conflict of Interest Statement

The authors declare that the research was conducted in the absence of any commercial or financial relationships that could be construed as a potential conflict of interest.
