# Supplementary material for: Characterization of culturable bacterial endophytes and their capacity to promote plant growth from plants grown using organic or conventional practices
Source: Front Plant Sci. 2015 Jul 10;6:490. doi: 10.3389/fpls.2015.00490 (PMC4498380; doi:10.3389/fpls.2015.00490)
Supplement: Supplementary file 1 [file Data_Sheet_1.DOCX]

SUPP Table 1 Table of taxonomic units assessed for impact on tomato growth as measured by an increase in height. Table includes species/genotypes, GenBank ID number, Phylum, p-value, height measurement and standard deviation from the mean of three replications. * indicates a significant height increase compared to the mock control.

SUPP Table 2 Table of taxonomic units assessed for impact on tomato growth as measured by an increase in fresh weight. Table includes species/genotypes, GenBank ID number, Phylum, p-value, weight measurement and standard deviation from the mean of three replications. * indicates a significant weight increase compared to the mock control.

SUPP Table 3 Table of taxonomic units assessed for impact on tomato growth as measured by an increase in dry weight. Table includes species/genotypes, GenBank ID number, Phylum, p-value, weight measurement and standard deviation from the mean of three replications. * indicates a significant weight increase compared to the mock control.
